# Case report: Effectiveness of low-dose methotrexate monotherapy in post-essential thrombocythemia myelofibrosis

**DOI:** 10.3389/fmed.2024.1285772

**Published:** 2024-04-18

**Authors:** Sebastian Francis, Tom King, Martin P. Zeidler

**Affiliations:** ^1^Department of Haematology, Royal Hallamshire Hospital, Sheffield, United Kingdom; ^2^Department of Dermatology, Royal Hallamshire Hospital, Sheffield, United Kingdom; ^3^The Bateson Centre and the School of Biosciences, The University of Sheffield, Sheffield, United Kingdom

**Keywords:** case report, methotrexate, psoriasis, myelofibrosis, quality of life

## Abstract

JAK/STAT pathway signalling is associated with both chronic inflammatory conditions such as psoriasis and haematological malignancies such as the myeloproliferative neoplasms (MPNs). Here we describe a 73yo female patient with a history of chronic plaque psoriasis, post-essential thrombocythemia myelofibrosis (MF) and a quality of life substantially impacted by both conditions. We report that 15 mg oral Methotrexate (MTX) weekly as a monotherapy is well tolerated, provides a substantial clinical improvement for both conditions and significantly improves quality of life. We suggest that the recently identified mechanism of action of MTX as a JAK inhibitor is likely to explain this efficacy and suggest that repurposing MTX for MPNs may represent a clinical- and cost-effective therapeutic option.

## 1 Introduction

JAK/STAT pathway signalling is central to multiple biological processes including inflammation, haematopoiesis and regulation of the immune system (1). Ectopic pathway activation is also associated with both chronic inflammatory conditions such as rheumatoid arthritis and psoriasis, as well as the chronic myeloid cancers collectively termed myeloproliferative neoplasms (MPNs) (2). MPNs include essential thrombocythemia (ET), associated with increased platelet counts, polycythemia vera (PV) associated with increased erythrocytes and myelofibrosis where bone marrow is replaced by fibrotic scar tissue—a condition that can be both a primary disease and a secondary development following ET or PV. In all cases, MPNs are associated with increased JAK/STAT pathway activity driven by gain-of-function mutations such as JAK2 V617F, indel mutations resulting in a +1 frameshift affecting the C-terminal of Calreticulin or activating mutations adjacent to the transmembrane region of the thrombopoietin receptor MPL W515L/K [reviewed in Morales et al. (2)].

Given the pathological role of JAK/STAT signalling in both inflammatory diseases and MPNs, pathway inhibitors targeting JAK tyrosine kinases have been developed and deployed to the clinic (3, 4). In inflammatory diseases kinase inhibitors such as Tofacitinib (JAK1/2), Baricitinib (JAK1/3) and the JAK1 inhibitors Abrocitinib, Itacitinib, Filgotinib and Upadacitinib have proven effective (4, 5) with the TYK2 inhibitor Deucravacitinib being licenced for chronic plaque psoriasis in 2023 [(6), for review see Shawky et al. (7)]. For MPNs, the JAK1/2 inhibitor Ruxolitinib and the JAK2 inhibitor Fedratinib have been approved for all MF patients and PV patients refractory to first line therapies (8).

In chronic plaque psoriasis topical corticosteroids, vitamin D analogues and phototherapy are used as first and second line therapies, with systemic treatments such as methotrexate, ciclosporin, acitretin and biologics used in more severe cases (9). First line therapies for MPNs include phlebotomy and aspirin to reduce thrombotic risk, broad spectrum anti-neoplastics such as hydroxy carbamide to reduce the proliferation of mutant stem cells/progenitors and pegylated α-Interferon. Although the precise mechanism-of-action of α-Interferon in MPNs is unclear, it is a potent JAK1/STAT1 ligand, a pathway associated with viral responses and cancer immunosurveillance (10).

We recently identified a novel activity for the anti-folate methotrexate (MTX) as an inhibitor of JAK/STAT pathway signalling in both *Drosophila* and human erythroid leukaemia-derived cells (11) and subsequently demonstrated efficacy in a mouse-based MPN model (12) and in an observational patient study (13)—results that have also been independently replicated by other groups (14). Originally developed as a chemotherapy agent in the 1950’s, by the 1980’s MTX had been repurposed at much lower doses as an anti-inflammatory and immuno-modulator in rheumatoid arthritis, psoriasis and other auto-immune diseases (15). Given that the mechanism-of-action of low dose MTX is unlikely to be mediated by folate pathway inhibition, we suggest that the anti-inflammatory mechanism of MTX is caused by its inhibition of JAK/STAT signalling (16)—a hypothesis backed up by both transcriptomic (17) and proteomic (18) analysis of MTX treated rheumatoid arthritis patients. However, despite the available pre-clinical data, clinical studies to directly test MTX in MPNs have yet to be launched—with a lack of commercial possibilities associated with repurposing a low-cost generic drug a factor in this delay.

Here we report a case where a Sheffield-based patient with chronic plaque psoriasis and recently developed post ET-MF was successfully treated with low-dose MTX monotherapy. A treatment which has significantly improved dermatological, haematological and quality of life measures at very modest cost.

## 2 Case description

The patient is a 73 year old female with a history of chronic plaque psoriasis previously treated with phototherapy as well as topical corticosteroid creams such as 0.1% w/w mometasone furoate, with a Psoriasis Area and Severity Index (PASI) of 12.9 and a Dermatology Life Quality Index (DLQI) of 27 (out of 30). The patient had previously been referred to district general hospital haematology in 2014 with thrombocytosis (PLT 540 × 10^9^/L) where then-available genetic testing for JAK2 and CALR did not identify any mutations.

In March 2021, the patient presented with pruritis and fatigue initially attributed to psoriasis. Although PLT < 600 × 10^9^/L, a subsequent blood film in June 2021 identified leucocytosis with teardrop erythrocytes indicative of splenic haematopoiesis and hence bone marrow fibrosis. Continuing night sweats and pruritis reported in July 2021 were followed by a bone marrow biopsy in September 2021 which confirmed marrow fibrosis (reticulin grade 2 out of 3) and a molecular identification via a myeloid NGS panel of MPL (NM 005373.2) c.1544G>T. p.Trp 515 Leu at VAF 0.784 and SF3B1 (NM 012433.2) c.1998G>C p. Lys 666 Asn at VAF 0.445. This led to a diagnosis of myelofibrosis—likely to be transformation from ET originally referred in 2014.

**Figure 1:**
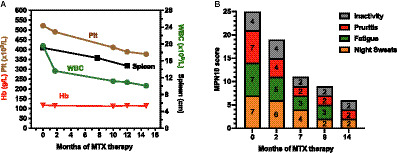
Changes in the indicated haematological **(A)** and constitutional **(B)** symptoms over the indicated timeframe since the beginning of methotrexate treatment.

At the beginning of treatment (month 0) the patient had haemoglobin (Hb) of 118 g/L, a white blood cell count (WBC) of 19.6 × 10^9^/L, PLT at 522 × 10^9^/L and splenomegaly of 19.1 cm length by ultrasound (Figure 1A). In addition, the patient recorded an MPN10 score of 25-scoring 7/10 for night sweats, pruritis and fatigue and 4/10 activity inactivity (Figure 1B).

## 3 Diagnostic assessment

Following consultation between dermatology and haematology, it was concluded that pegylated α-interferon as a treatment for MF was unsuitable given the potential for adverse drug interactions with immunosuppressive therapies planned for the treatment of plaque psoriasis. Given its authorisation as a treatment for plaque psoriasis and its potential as a JAK-inhibitor with activity in MPNs (11–13), the patient was started on MTX 15 mg weekly, with 5 mg folic acid on days 2 and 3 after the MTX dose. MTX was primarily prescribed for the plaque psoriasis indication at a dose based on NICE guidelines (9). It has been well tolerated, liver function remains within normal range (Alanine transaminase 29 U/L [Normal range: 0–33]) and has not been associated with adverse events (19).

Over the course of the next 15 months, haematological values progressively improved with WBC reducing to 10.1 × 10^9^/, PLT to 377 × 10^9^/L whilst haemoglobin remained largely unchanged (Figure 1A)—suggesting that MTX is not causing general myelosuppression. Spleen length reduced from 19.1 to 14.8 cm (Figure 1A)—a change in length of 22% which equates to an approximate 47% reduction in overall spleen volume (assuming spleen diameter has decreased proportionally). Simultaneously, MPN10 scores markedly reduced over the same timeframe with all reported constitutional symptoms consistently improving (Figure 1B). Molecularly, the mutant MPL variant allele fraction decreased marginally from 0.784 at diagnosis to 0.762 after 20 months of MTX.

Dermatologically, the patients skin also improved with the area and severity of psoriasis (PASI) improving from 12.9 to 0 (completely clear skin) after 4 months of treatment, whilst DLQI improved from 27 to 1 after 9 months.

## 4 Discussion

Here we present a report of a patient with chronic plaque psoriasis and ET-derived secondary MF with a poor quality of life. We find that 15 mg weekly of MTX – a typical starting dose in psoriasis (9)—is sufficient to control both dermatological and MF symptoms whilst providing highly effective symptomatic control (Figure 1). This outcome is consistent with our previous identification of MTX as a JAK inhibitor (11), and the central role for JAK/STAT signalling in mediating both inflammatory processes and haematological disease (1). The therapy is well tolerated and has not caused any adverse events over >15 months. However, despite this individual outcome, it should be noted that low-dose MTX is associated with side effects—including gastrointestinal upset and hepatotoxicity as well as rarer cases of myelosuppression and myelodysplastic syndrome. As such, supplementation with folic acid to minimise GI effects, liver function testing and monitoring of blood counts was undertaken in line with Psoriasis management guidelines (9).

Interestingly, in this case MTX has provided a consistent but relatively gradual response in both haematological and MPN10 scores, with a slightly faster response as gauged by dermatological measures. This response is however slower than that generally produced by the JAK1/2 inhibitor Ruxolitinib where responses have been documented within 8–24 weeks (8). Whilst the reason for this difference is not immediately clear, a similar longer term therapeutic profile is also observed when MTX is used to treat rheumatoid arthritis (20) and may stem from the time taken for intracellular polyglutamated MTX to build up *in vivo*, and/or the time taken for existing inflammation to resolve. This longer timeframe, building over the span of several months, suggests that long term therapy is likely to bring progressively greater benefits and that a modest response in the short term is not necessarily an indication of therapeutic failure.

Given our premise that the effectiveness of low-dose MTX reported here is likely a result of JAK inhibition, it is relevant to compare MTX to targeted JAK inhibitors such as Tofacitinib, Baricitinib, Abrocitinib, Itacitinib, Filgotinib, Upadacitinib and Deucravacitinib used to treat inflammatory disease as well as Ruxolitinib and Fedratinib used in MPNs [see (7) for review]. Since 2011 when it was launched as the first clinically licenced JAK inhibitor, Ruxolitinib has become a mainstay treatment for MF patients and PV patients with an inadequate response to conventional therapies—often delivering significant improvements in haematological, splenic and constitutional symptoms (8). However, subsequent experience suggests that JAK inhibition does not routinely reduce driver mutation alleleic burden and has only a modest impact on overall survival (21). Furthermore, in the longer term many patients develop resistance/intolerance to the drug—a change with generally poor prognosis (22). In addition, first generation JAK inhibitors such as Tofacitinib have also been associated with sometimes serious cardiovascular, oncological, and venous thromboembolism complications (23) prompting the EMA to issue specific guidance to minimise these risks (EMA/142279/2023). It remains to be seen if more recently introduced second generation inhibitors will be able to avoid similar undesired effects.

Although JAK inhibition is of undoubted clinical value, the relatively recent introduction of many of these inhibitors mean that most are still on-patent and consequently associated with high drug costs. As such, the benefits of JAK inhibition are largely restricted to patients in developed countries with well-funded healthcare systems. As a result, a substantial proportion of the global population lack access to current JAK inhibitor treatment options. Given these factors, our identification of MTX as both a JAK inhibitor (11, 12) and an effective monotherapy in post-ET MF suggests that this low cost, WHO ‘essential drug’ could potentially be repurposed to provide effective and safe control of haematological and symptomatic aspects of MPNs. Considering previous observational reports that also suggest effectiveness in MPN patients (13, 14), we suggest that formal larger scale Phase II/III trials to determine the wider potential for the repurposing MTX in the treatment of MPNs are urgently needed.

## 5 Patient perspectives

When asked to provide a comment for this manuscript the patient stated that ‘my skin is amazing (…). I feel very well on this medication, and it has changed my life. I do not have to hide behind my terrible plaques (…)’.

## Data availability statement

The original contributions presented in the study are included in the article material, further inquiries can be directed to the corresponding author.

## Ethics statement

Ethical approval was not required for the study in accordance with the local legislation and institutional requirements. Written informed consent was obtained from the individual for the publication of data included in this article.

## Author contributions

SF: Writing – review & editing, Investigation, Data curation. TK: Writing – review & editing, Investigation, Data curation. MZ: Writing – review & editing, Writing – original draft, Visualization, Data curation, Data analysis.
